# Characterization of a *Lactobacillus brevis* strain with potential oral probiotic properties

**DOI:** 10.1186/s12866-018-1369-3

**Published:** 2018-12-22

**Authors:** Fang Fang, Jie Xu, Qiaoyu Li, Xiaoxuan Xia, Guocheng Du

**Affiliations:** 1 Key Laboratory of Industrial Biotechnology, Ministry of Education, School of Biotechnology, Jiangnan University, Wuxi, 214122 China; 2 State Key Laboratory of Food Science and Technology, Jiangnan University, Wuxi, 214122 China; 3 The Key Laboratory of Carbohydrate Chemistry and Biotechnology, Ministry of Education, Jiangnan University, Wuxi, 214122 China

**Keywords:** *Lactobacillus brevis*, Antimicrobial activity, Hydrogen peroxide, Adhesion, Immunomodulation

## Abstract

**Background:**

The microflora composition of the oral cavity affects oral health. Some strains of commensal bacteria confer probiotic benefits to the host. *Lactobacillus* is one of the main probiotic genera that has been used to treat oral infections. The objective of this study was to select lactobacilli with a spectrum of probiotic properties and investigate their potential roles in oral health.

**Results:**

An oral isolate characterized as *Lactobacillus brevis* BBE-Y52 exhibited antimicrobial activities against *Streptococcus mutans*, a bacterial species that causes dental caries and tooth decay, and secreted antimicrobial compounds such as hydrogen peroxide and lactic acid. Compared to other bacteria, *L. brevis* BBE-Y52 was a weak acid producer. Further studies showed that this strain had the capacity to adhere to oral epithelial cells. Co-incubation of *L. brevis* BBE-Y52 with *S*. *mutans* ATCC 25175 increased the IL-10-to-IL-12p70 ratio in peripheral blood mononuclear cells, which indicated that *L. brevis* BBE-Y52 could alleviate inflammation and might confer benefits to host health by modulating the immune system.

**Conclusions:**

*L. brevis* BBE-Y52 exhibited a spectrum of probiotic properties, which may facilitate its applications in oral care products.

## Background

Oral infectious diseases, such as dental caries and periodontal diseases, represent one of the most common infections that severely impact human health [1]. As a result of antibiotics overuse and misuse and the emergence of antibiotic-resistant strains, probiotic therapy has been gradually applied to prevent and alleviate infectious diseases [2–4]. *Lactobacillus* is one of the main probiotic genera that has been widely studied for its potential roles on oral health. Evidence shows that consumption of milk enriched with *Lactobacilllus rhamnosus* GG decreases the risk of dental caries in children [5]. Similarly, *Lactobacillus reuteri* reduces the risk of gingivitis [6]. Therefore, lactobacilli with probiotic properties may prevent the colonization of oral pathogens through different mechanisms.

Probiotic strains produce antimicrobial components such as hydrogen peroxide and lactic acid, which inhibit the growth of oral pathogens (e.g., *Streptococcus mutans*). However, excessive acid production by lactobacilli may result in the demineralization of the tooth enamel and contribute to dental caries [7]. Moreover, auto-aggregation of the same strain, co-aggregation with common oral strains, and production of extracellular polysaccharides by probiotics favor the formation of biofilms, which protect bacterial strains against environmental changes and contribute to cell aggregation and colonization [8, 9]. The adhesion of strains to oral epithelial cells prevents the colonization of pathogens [10]. The modulation of the immune system by probiotics, which affects human health, is assessed by the release of anti-inflammatory cytokine interleukin (IL)-10 and pro-inflammatory cytokine IL-12p70. Strains with high IL-10-to-IL-12p70 ratios confer benefits to human health [11].

The aim of this study was to characterize *Lactobacillus* strains with potential probiotic properties including production of antimicrobial compounds and extracellular polysaccharides and the capability to adhere to oral epithelial cells.

## Results

### Isolation of lactobacilli with antimicrobial activity

**Fig. 1:**
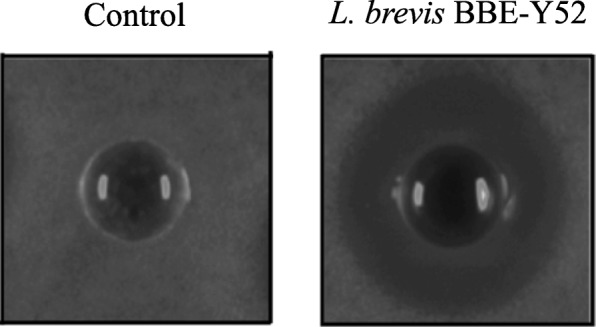
Antimicrobial activity of *Lactobacillus brevis* BBE-Y52 against *Streptococcus mutans* ATCC 25175. Control, growth of *S*. *mutans* ATCC 25175 with addition of lactic acid (pH 4.2) in the well; *L*. *brevis* BBE-Y52, inhibition of *S*. *mutans* ATCC 25175 by the supernatant of *L*. *brevis* BBE-Y52

We analyzed 32 oral isolates. Among them, one *Lactobacillus* strain isolated from dental plaque exhibited antimicrobial activities against *S*. *mutans* ATCC 25175 (Fig. 1). This strain was identified with 16S rRNA gene and *pheS* sequence blast and designated *L. brevis* BBE-Y52. In addition to bacteriocin, hydrogen peroxide is another antimicrobial compound that inhibits bacterial growth. Production of hydrogen peroxide is rare in *Lactobacillus* due to their weak tolerance to oxygen. However, *L. brevis* BBE-Y52 produced 0.06–0.15 mM hydrogen peroxide. Lactic acid is another antimicrobial compound that is produced by almost all lactobacilli. Production of lactic acid is necessary for the inhibition of oral pathogens; however, excessive acid production may result in tooth decay. Therefore, we measured the capacity of *L. brevis* BBE-Y52 to produce acid and compared it to other lactic acid bacteria. We observed that *L. brevis* BBE-Y52 was a weak acid producer compared with *Lactobacillus salivarius* T1 and *S*. *mutans* ATCC 25175, based on the difference in average pH values when cultivated in MRS broth supplemented with various carbon sources (Table 1). Moreover, the final concentration of lactic acid from *L. brevis* BBE-Y52 (1.74 ± 0.12 g/L) in the broth was lower than that from *L*. *salivarius* T1 and *S*. *mutans* ATCC 25175 (2.64 ± 0.09 and 2.53 ± 0.07 g/L, respectively).

**Table 1:** Acid production by *Lactobacillus brevis* BBE-Y52, *Lactobacillus salivarius*, and *Streptococcus mutans*

Strains	pH^a^				Average pH^b^	Lactic acid^c^ (g/L)
	Glucose	Sucrose	Lactose	Fructose		
*L. brevis* BBE-Y52	3.86 ± 0.01	4.02 ± 0.03	4.07 ± 0.02*	3.77 ± 0.03	3.93*	1.74 ± 0.12**
*L*. *salivarius* T1	3.81 ± 0.03	3.90 ± 0.04	3.98 ± 0.02	3.80 ± 0.02	3.87	2.64 ± 0.09
*S*. *mutans* ATCC 25175	3.90 ± 0.02*	3.87 ± 0.03*	3.95 ± 0.01	3.74 ± 0.03	3.86	2.53 ± 0.07

^a^, pH values of strain cultures grown in medium containing single carbon source; ^b^, average pH values; ^c^, production of lactic acid by corresponding strains cultivated in MRS broth

* and ** represent significant (*P* < 0.05) and extremely significant (*P* < 0.01) differences based on Student’s t-test

### *L. brevis* BBE-Y52 susceptibility to lysozyme, hydrogen peroxide, and antibiotics

**Fig. 2:**
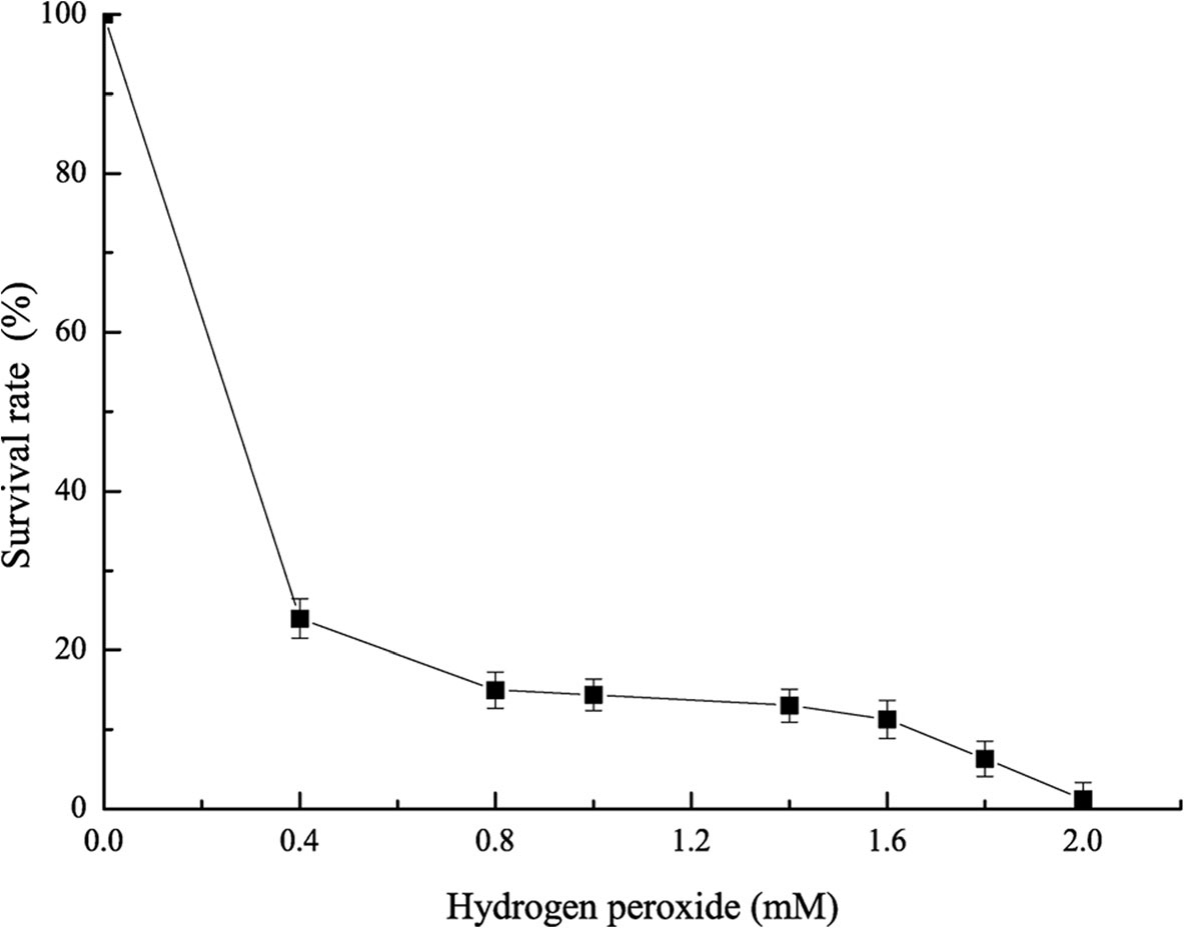
Tolerance of *Lactobacillus brevis* BBE-Y52 to hydrogen peroxide

Microorganisms in the oral cavity may be affected by lysozyme and hydrogen peroxide. We measured the *L. brevis* BBE-Y52 resistance to lysozyme and hydrogen peroxide. *L. brevis* BBE-Y52 showed tolerance to 1.0 mg/mL lysozyme, which is much higher than that present in the oral cavity [12]. The *L. brevis* BBE-Y52 tolerance to hydrogen peroxide was 2.0 mM (Fig. 2). *L. brevis* BBE-Y52 was resistant to kanamycin and streptomycin and susceptible to ampicillin, tetracycline, and chloramphenicol (Table 2).

**Table 2:** The tolerance of *L*. *brevis* BBE-Y52 to antibiotics

Antibiotics (μg/L)	1	2	4	8	16	32	64
ampicillin	–	–	–	–	–	–	–
chloramphenicol	+	+	–	–	–	–	–
tetracycline	+	+	+	–	–	–	–
kanamycin	+	+	+	+	+	+	+
streptomycin	+	+	+	+	+	+	+

+, tolerant; −, susceptible

### Evaluation of the aciduric profile of *L. brevis* BBE-Y52

**Fig. 3:**
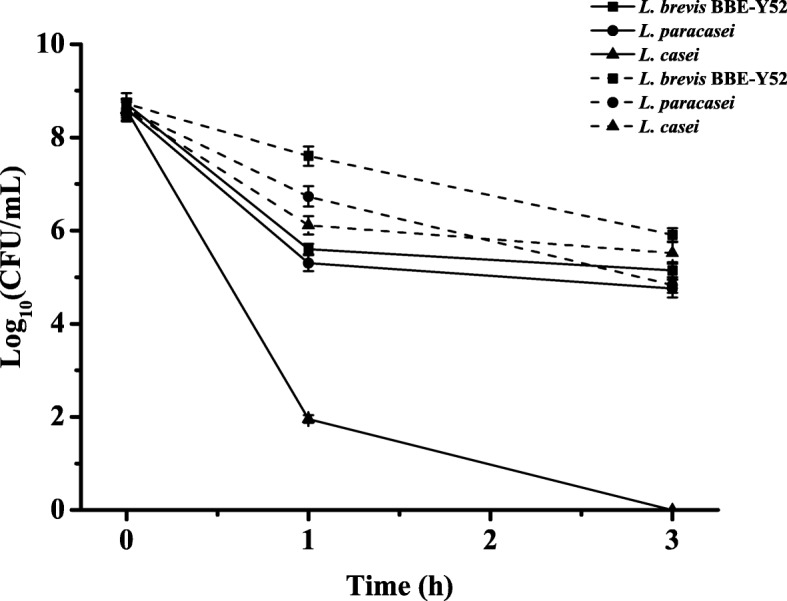
Aciduric profile of *Lactobacillus brevis* BBE-Y52. Survival of *Lactobacillus* strains challenged with acidic conditions at pH 2.0 (lines) and pH 3.0 (dot lines)

Resistance to acid stress is a very important trait for probiotics and may help them to survival in harsh niches and therefore expand their applications. In this study, resistance of *L. brevis* BBE-Y52 to acid stress was examined, and the aciduric profile of it was compared with that of two *Lactobacillus* strains (*L*. *casei* DSM 20011 and an oral isolate *L. paracasei* XJ02). As shown in Fig. 3, the survival rate of *L. brevis* BBE-Y52 is higher than that of *L*. *casei* DSM 20011 and *L. paracasei* XJ02 at both pH 2.0 and pH 3.0. After 3 h exposure to pH 2.0, no viable cell counts were determined for *L*. *casei* DSM 20011, while the survival rate for *L. brevis* BBE-Y52 and *L. paracasei* XJ02 were 0.027% (3.6 log cycles reduction in viable cell numbers) and 0.014%(3.9 log cycles reduction in viable cell numbers), respectively.

### Biofilm formation and adhesion of *L. brevis* BBE-Y52 to KB cells

Biofilms confer protection to microorganisms against environmental changes. Both EPS production and aggregation of bacteria contribute to biofilm formation. *L. brevis* BBE-Y52 formed viscous colonies and produced 106.6 mg/L EPS.

**Fig. 4:**
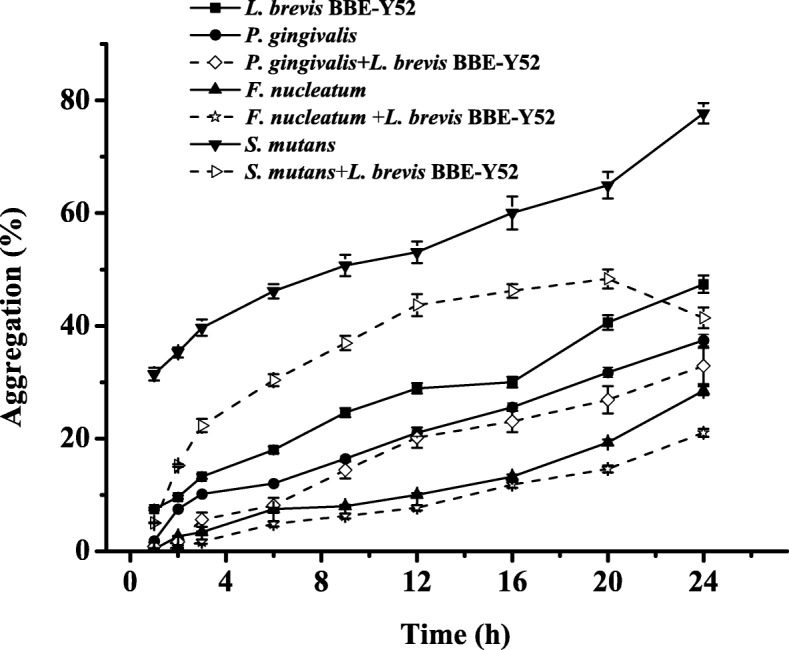
Auto-aggregation of *Lactobacillus brevis* BBE-Y52 and coaggregation of BBE-Y52 with oral pathogens. Auto-aggregation (lines) of individual strain and coaggregation (dot lines) of BBE-Y52 with oral pathogens. Solid symbols: square, *L*. *brevis* BBE-Y52; circle, *Porphyromonas gingivalis* GIM1.851; upper triangle, *Fusobacterium nucleatum* CGMCC 1.2528; lower triangle, *Streptococcus mutans* ATCC 25175; Open symbols: right triangle, *L*. *brevis* BBE-Y52 + *S*. *mutans* ATCC 25175; diamond, *L*. *brevis* BBE-Y52 + *P. gingivalis* GIM1.851; star, *L*. *brevis* BBE-Y52 + *F*. *nucleatum* CGMCC 1.2528

**Fig. 5:**
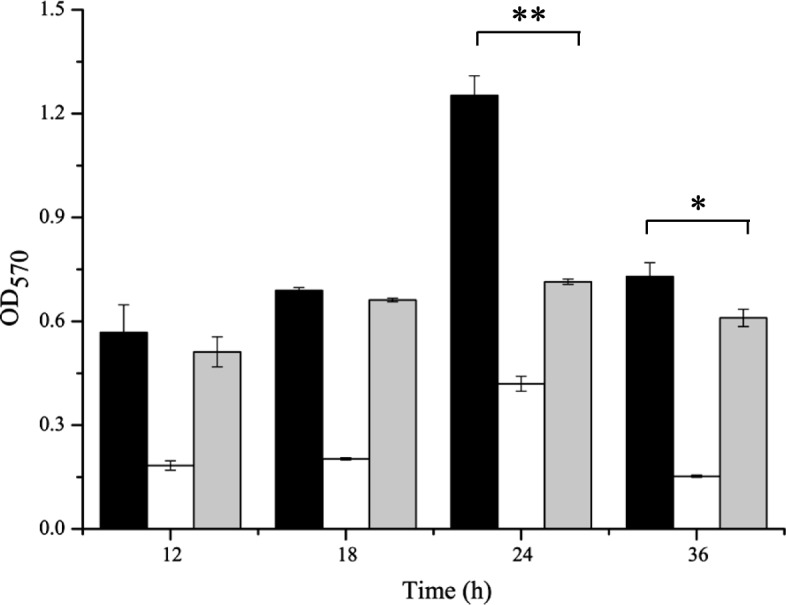
Biofilm formation by *Lactobacillus brevis* BBE-Y52 and *S*. *mutans* ATCC 25175. Black bars, white bars and grey bars represent the biofilm formed by individual *Streptococcus mutans* ATCC 25175, *L*. *brevis* BBE-Y52, and both strains, respectively. * and ** represent significant (*P* < 0.05) and extremely significant (*P* < 0.01) differences based on Student’s t-test

The ability of *L. brevis* BBE-Y52 to auto-aggregate and aggregate with other bacteria was investigated and compared with that of oral pathogens including *Streptococcus mutans*, *Porphyromonas gingivalis* and *Fusobacterium nucleatum*. All tested strains exhibited a capacity to auto-aggregate after 3 h incubation (Fig. 4). The *L. brevis* BBE-Y52 auto-aggregation capacity was higher than that of all tested strains except *S*. *mutans* ATCC 25175. *L. brevis* BBE-Y52 exhibited co-aggregation with all tested strains, reduced *S*. *mutans* ATCC 25175 aggregation significantly, and slightly reduced *P*. *gingivalis* GIM 1.851 and *F. nucleatum* CGMCC 1.2528 aggregation. Formation of mature biofilm by *L. brevis* BBE-Y52 and *S*. *mutans* ATCC 25175 was observed following 24 h incubation. *S*. *mutans* ATCC 25175 exhibited a stronger capacity for biofilm formation than *L. brevis* BBE-Y52. Co-culture of *L. brevis* BBE-Y52 with *S*. *mutans* ATCC 25175 resulted in a reduction of biofilm formation by the pathogen (Fig. 5).

Colonization in the oral cavity is an important property for a probiotic strain. We evaluated the adhesion of probiotic strains to the human oral epithelial cell line KB using the quantitative (viable adherent cell counting) method. *L. brevis* BBE-Y52 exhibited a capacity to adhere to KB cells. The ratio of viable adherent *L. brevis* BBE-Y52 cells to KB cells was 15 ± 2: 1.

### *L. brevis* BBE-Y52 effects on cytokine production by human PBMCs

Immunomodulation is an important mechanism that affects human health. Strains with immunomodulatory properties are desirable. Therefore, *L. brevis* BBE-Y52 immunomodulatory properties were evaluated by measuring the release of cytokines, including pro-inflammatory IL-12p70 and anti-inflammatory IL-10. In the model of cellular immunity analysis, *S*. *mutans* ATCC 25175 caused inflammation in PBMCs, indicated by the decreased IL-10-to-IL-12p70 ratio. Co-culturing of PBMCs with *L. brevis* BBE-Y52 increased IL-10 levels and decreased the IL-10-to-IL-12p70 ratio (Table 3). In *S*. *mutans* ATCC 25175-inflamed PBMCs, *L. brevis* BBE-Y52 significantly increased the IL-10-to-IL-12p70 ratio from 4.1 to 3.8, suggesting that *L. brevis* BBE-Y52 could alleviated inflammation in PBMCs (Table 3).

**Table 3:** PBMC cytokine levels in response to co-culturing with *L*. *brevis* BBE-Y52 or *S*. *mutans*

Cytokine	^a^PBMCs			^b^PBMCs + *S*. *mutans*	
	Control	BBE-Y52	*S*. *mutans*	Control	BBE-Y52
IL-10 (pg mL^− 1^)	164 ± 2	186 ± 2	177 ± 4	248 ± 2	243 ± 3
IL-12p70 (pg mL^− 1^)	28 ± 2	31 ± 1	33 ± 1	65 ± 2	59 ± 2
IL-10/IL-12p70	5.8 ± 0.2	6.1 ± 0.1*	5.4 ± 0.1*	3.8 ± 0.1	4.1 ± 0.1*

^a^, PBMCs incubated either alone (control) or with BBE-Y52/*S*. *mutans* for 12 h; ^b^, *S*. *mutans* treated PBMCs was incubated either alone (control) or with BBE-Y52 for 24 h; * IL-10-to-IL-12p70 ratio was significant (*P* < 0.05) based on Student’s t-test

## Discussion

Lactobacilli with probiotic properties have been used in clinical trials due to their role in oral health [13–16]. *Lactobacillus crispatus*, *Lactobacillus fermentum*, and *Lactobacillus gasseri* (oral isolates) and *L. rhamnosus* LGG (a non-oral isolate) have oral probiotic properties including reduction of oral pathogens counts and adherence to oral epithelial cells [17, 18]. In this study, *L. brevis* BBE-Y52, a strain isolated from the dental plaque of a healthy donor, exhibited oral probiotic properties such as inhibition of the oral pathogen species *S*. *mutans*, reduction of aggregation of *S*. *mutans*, *P*. *gingivalis* and *F*. *nucleatum*, production of hydrogen peroxide, resistant to acidic condition, adherence to KB cells, and alleviation of inflammation. This is the first time that a *Lactobacillus* strain is shown to have so many composite oral probiotic properties, which indicating the potential applications (inhibition or exclusion of oral pathogens, persistence in oral cavity, anti-inflammation) of *L. brevis* BBE-Y52 in oral care products.

It has been shown that a few *Lactobacillus* strains exhibited capability of inhibition oral pathogens. *L. rhamnosus* LGG, a human probiotic, reduces the counts of oral pathogens *Streptococcus sanguinis* and *Candida albicans* and reduces the biofilm-forming ability of *Fusobacterium nucleatum*, an oral bacterium associated with periodontal disease [18]. Mishra et al. demonstrated that a probiotic rinse (containing *Streptococcus*) was as effective as a chlorhexidine digluconate rinse in reducing *C. albicans* counts [19]. *Lactobacillus* strains isolated from the oral cavity showed antibacterial activity against the periodontal pathogen *Porphyromonas gingivalis*, but they did not inhibit the growth of *Prevotella intermedia* or *S*. *mutans* when the acid effect was removed [17]. Lactic acid produced by lactic acid bacteria inhibits the growth of Gram-negative bacteria including the oral pathogen *P*. *gingivalis* [20]. In this study, the antimicrobial activity of *L. brevis* BBE-Y52 against *S*. *mutans* was confirmed after removing the lactic acid effect (Fig. 1). This probiotic might inhibit the growth of other oral pathogens. However, lactic acid may potentially weaken the tooth enamel [7]. Therefore, a weak lactic acid producer is more suitable for oral care products. Compared to other *Lactobacillus* strains tested in this study and those previously reported (*Lactobacillus acidophilus* CRL 1259 and *Lactobacillus* strain L1 and L2 produce 8.57–9.28 and 8.85 g/L lactic acid, respectively) [21, 22]*, L. brevis* BBE-Y52 is a weak lactic acid producer. Moreover, *L. brevis* BBE-Y52 survives better at pH 2.0 and pH 3.0 than other two tested *Lactobacillus* strains (Fig. 3). After 3 h treatment at pH 2.0, the viable cell number of *L. brevis* BBE-Y52 is 3.6 log cycles reduction, this is much better than that of 90% probiotic *Lactobacillus* strains (1.5–9.0 log cycles reduction) [23]. Hydrogen peroxide is another antimicrobial substance that can be produced by streptococci and some commensal lactobacilli [24]. *L. brevis* BBE- Y52 produced up to 0.15 mM hydrogen peroxide when grown in MRS broth in microaerobic conditions. This is lower than that of the intestine isolate *Lactobacillus johnsonii* NCC 533, which produces 1 mM hydrogen peroxide when cultivated in LAPTg medium under aerobic conditions [24]. *L. brevis* BBE- Y52 is competitive, because *L*. *johnsonii* NCC 533 hardly produces any hydrogen peroxide in anaerobic conditions [24].

In this study, the co-aggregation with *L. brevis* BBE-Y52 decreased *S*. *mutans P. gingivalis* and *F. nucleatum* aggregation (Fig. 4), which may in part reduce the risk of dental caries and periodontal disease. The capacity to co-aggregate with oral microbes and form biofilms may contribute to *L. brevis* BBE-Y52 colonization in the oral cavity. In addition, the adherence ratio of *L. brevis* BBE-Y52 to KB cells was (15 ± 2): 1, which demonstrated that *L. brevis* BBE-Y52 has the capacity to adhere to KB cells, based on classification criteria [25]. Saeed and Heczko demonstrated that lactobacilli with high auto-aggregation are highly adherent to the KB cell line [26], consistent with our findings. *Lactobacillus* strains of oral origin adhered to oral epithelial cells with an adherence of 60 bacterial cells/mm^2^ for HO and/or HSC cells [17]. According to MIC analysis, *L. brevis* BBE- Y52 is resistant to kanamycin and streptomycin, but susceptible to ampicillin, chloramphenicol and tetracycline. In contrast, *Lactobacillus plantarum* and *Lactobacillus oris* strains are resistant to tetracycline [27, 28]. *L. brevis* BBE-Y52 tolerates 1 mg/mL lysozyme, some oral lactobacilli tolerate up to 10 mg/mL lysozyme [28], while *L. plantarum* strains tolerate up to 0.1 mg/mL lysozyme [29]. Immunomodulation by probiotics is an important aspect that confers benefits on human health. Immunomodulation can be evaluated by the release of cytokines as key regulators. Following co-incubation of inflamed PBMCs (by *S*. *mutans*) with *L. brevis* BBE-Y52, inflammation subsided based on the increase in IL-10-to-IL-12p70 ratio (Table 2).

## Methods

### Strains and growth conditions

*Lactobacillus* strains were grown in de man, Rogosa, Sharpe (MRS, Oxoid, Hampshire, UK) medium and incubated at 37 °C under microaerophilic conditions (5% CO_2_) for 24–48 h. *S. mutans* ATCC 25175 was grown in brain heart infusion medium (BHI, Oxoid, Hampshire, UK) at 37 °C under microaerophilic conditions. *Porphyromonas gingivalis* GIM1.851 and *Fusobacterium nucleatum* CGMCC 1.2528 were grown at 37 °C anaerobically in TSB medium and PYG medium, respectively.

### Lactobacilli isolation and identification from human saliva and dental plaques

Human saliva and dental plaques samples of 10 healthy donors (five female and five male; mean age 24 ± 3 years) were obtained from Wuxi No. 2 People’s Hospital (Wuxi, Jiangsu Province, China) according to the method reported by Krajden [30]. *Lactobacillus* strains were isolated by plating the serial diluted samples on MRS agar and cultivated at 37 °C under microaerophilic conditions. Genomic DNA of *Lactobacillus* strains were extracted using EZNA genomic DNA isolation kits (Omega Bio-Tek, Doraville, USA). Strains were identified by analysis of 16S rDNA (amplified by primers 16SF 5′-TACGGYTACCTTGTTACGACTT-3′ and 16SR 5′-AGAGTTTGATCMTGGCTCAG-3′) and *pheS* (amplified by primers pheSF 5′-TTCCCATTTACGGAGCCTTCTG-3′ and pheSR 5′-GCACCATACCGGCACCTAACAC-3′) sequences and blasted against the NCBI database.

### Antimicrobial activity assay

Antimicrobial activities of isolates against *S. mutans* ATCC 25175 were evaluated by the agar well-diffusion method [31]. Briefly, 10 mL of base agar (1.5%, *w*/*v*) was plated on a Petri dish and allowed to cool before placing on sterile Oxford Cups. *S. mutans* ATCC 25175 (final concentration in agar: 10^6^ CFU/mL) was mixed with 10 mL of semi-solid BHI agar (0.8%, w/v) and overlaid on the base agar. Oxford Cups were removed when the upper layer set. Finally, 100 μL of supernatant of bacterial culture or heat-inactivated supernatant (control) was added to wells and incubated at 37 °C (5% CO_2_) for 24 h. The inhibition zones represent the growth inhibition of *S. mutans* ATCC 25175 by corresponding strains.

### Analysis of acid production

*Lactobacillus* strains and *S*. *mutans* were inoculated in MRS broth (devoid of carbon source) supplemented with single carbon source (equal moles of glucose, sucrose, lactose, and fructose) and grown at 37 °C and 5% CO_2_ for 24 h. Following a 24-h incubation, the pH value of the broth was measured. Production of lactic acid by these strains grown in MRS at 37 °C (5% CO_2_) for 24 h was determined with high performance liquid chromatography using a modified method [32]. Strains were removed by centrifugation at 10,000 rpm for 10 min. Corresponding supernatants were passed through a 0.22-μm membrane filter, and 20 μL of the filtrate or its dilution was injected onto HPLC for lactic acid quantification. Separation was performed using an HPLC organic acid analysis column (300 mm × 7.8 mm, BIO-RAD, Hercules, CA, USA) at 40 °C, using 0.05 mM sulfuric acid as the mobile phase (flow rate 0.6 mL/min, 30 min). Lactic acid was detected at 210 nm.

### Detection of acid resistance of *Lactobacillus*

*L. brevis* BBE-Y52, *L*. *casei* DSM 20011 and *L. paracasei* XJ02 (a strain isolated from oral cavity, this study) were grown in MRS broth at 37 °C. Cells of these strains collected during the exponential growth phase were harvested by centrifugation (8000 rpm, 5 min), and then washed three times with phosphate buffer saline (PBS, pH 7.2) before suspended in PBS or PBS adjusted to pH 2.0 and 3.0 using 5 M HCl, and incubated at 37 °C. Samples of each solution were taken at different time intervals, diluted and plated on MRS agar. Cell numbers were counted and used for calculating the survival rate.

### Detection of extracellular polysaccharides (EPS) and hydrogen peroxide

Bacterial strains were grown on MRS agar containing 10% (*w*/*v*) sucrose, and the plates were incubated at 30 °C for 48 h. Strains that appeared as viscous colonies were considered to be EPS producers [33]. For quantitative analysis of EPS, strains were grown at 30 °C for 48 h in MRS broth containing 10% (w/v) sucrose. Proteins in cultures were precipitated with trichloroacetic acid (Sigma–Aldrich, St. Louis, MO, USA) and stirred for 30 min on ice. Following centrifugation at 15,000 *g* for 30 min at 4 °C, cold ethanol (three times the volume) was added to the supernatant and stored overnight at 4 °C to precipitate EPS. The sediment was collected by centrifugation at 15,000 *g* for 30 min at 4 °C and dissolved in distilled water. EPS was determined by phenol–sulfuric acid method using D-glucose as a standard [34].

To measure hydrogen peroxide production, bacterial strains were grown in MRS broth and incubated at 37 °C (5% CO_2_) for 24 h. Hydrogen peroxide in cell-free supernatant of corresponding bacterial cultures was measured using semi-quantitative test strips (Merck, Poole, UK), which detect hydrogen peroxide at 0 to 25 mg/L.

### Lysozyme, hydrogen peroxide, and antibiotic susceptibility

The susceptibility of lactobacilli (10^7^ CFU/mL) to lysozyme (Thermo Fisher, Waltham, MA, USA) was examined by an agar well-diffusion method [28]. Susceptibility to hydrogen peroxide (Sigma–Aldrich) was evaluated by the survival of the corresponding strain when exposed to hydrogen peroxide [35]. The *Lactobacillus* strain was cultivated in MRS at 37 °C (5% CO_2_) for 24 h. Cells were harvested by centrifugation (8000 rpm, 15 min) and rinsed twice with phosphate-buffered saline (PBS, pH 7.0) before re-suspending (10^8^ CFU/mL) in PBS containing 0–2 mM hydrogen peroxide at 37 °C (5% CO_2_) for 1 h. Corresponding cultures were plated in MRS agar, and viable cells were counted and compared with the control (0 mM hydrogen peroxide). Survival rate was calculated to evaluate the resistance to hydrogen peroxide. Antibiotic susceptibility of lactobacilli was determined by the disk diffusion method [36]. Strains were classified as resistant or susceptible by comparing the minimum inhibitory concentrations of antibiotics (Sigma–Aldrich) with European Food Safety Authority standards [37].

### Aggregation assays

Bacterial aggregation of single strain (auto-aggregation) and two strains were examined [38, 39]. Cells of *S. mutans* ATCC 25175, *P*. *gingivalis* GIM1.851 and *F*. *nucleatum* CGMCC 1.2528 collected during the exponential growth phase were harvested, cells were washed twice with PBS, and suspended in PBS to an OD_600_ value of 0.5 (A_0_). Absorbance at 600 nm of the upper suspension (A_t_) was measured after 1–3 h incubation. Coaggregations of oral lactobacilli with the foregoing strains were determined by combining equal volumes (500 μL) of the adjusted suspensions of isolates (A_probio_) and oral pathogens (A_pat_). Coaggregation was calculated using the formula described by Collado [38].

Auto-aggregation (%) = (A_0_ − A_t_)/A_0_ × 100.

Coaggregation (%) = [(A_pat_ + A_probio_)/2 –(A_mix_)/ (A_pat_ + A_probio_)/2] × 100.

where A_mix_ represents absorbance at 600 nm of culture of two strains.

### Biofilm formation assays

The formation of biofilm by oral isolates and *S*. *mutans* ATCC 25175 was assessed using the microtiter plate method [40]. *Lactobacillus* strains and *S*. *mutans* were cultivated in MRS or BHI at 37 °C (5% CO_2_) for 24 h. Subsequently, 125 μL of cell suspensions (OD_600_ = 1.0) of individual strains were added to wells of a 24-well plate containing 875 μL BHI broth (750 μL BHI broth for two strains), and incubated at 37 °C (5% CO_2_) for 12–36 h. Cell suspensions were removed by careful pipetting. Wells were rinsed three times with PBS and air-dried for 30 min. Wells were stained with crystal violet (1%, *w*/*v*) for 20 min, rinsed with PBS, and air-dried for 30 min. Ethanol (1 mL, 95%) was added, and absorbance at 570 nm was measured.

### Adhesion assays

The oral epithelial carcinoma cell line (KB) was used to assess the adhesion of oral lactobacilli [41, 42]. KB cells (provided by Prof. Jian Jin, and purchased from ATCC, Manassas, VA, USA) were grown in minimum essential medium (MEM; Invitrogen, Carlsbad, CA, USA) supplemented with 10% (*v*/v) fetal bovine serum (Invitrogen) at 37 °C (5% CO_2_). A concentration of 3.0 × 10^5^ KB cells was seeded in 35-mm-diameter dishes (Corning, New York, NY, USA) and incubated until the formation of a complete monolayer. The KB cells monloayer was washed twice with PBS, and an aliquot of 2 mL minimum essential medium (MEM, Invitrogen) was added to the plates and incubated at 37 °C (5% CO_2_) for 30 min. The bacterial cultures were washed and suspended in MEM (10^8^ CFU/mL). Bacterial suspension (120 μL) was seeded onto the KB cells monolayer and incubated for 1 h at 37 °C (5% CO_2_). The KB cells monolayer were subsequently washed to remove unattached bacteria. Methanol (3 mL) was added to fix the cells (10 min) and removed prior to staining with 3 mL Giemsa stain for 30 min. For detecting the adhesion of lactobacilli to KB cells, the dishes were rinsed, dried at 37 °C for 16 h, and examined by microscopy [42]. To quantify the adhesion capability of bacteria to KB cells, the monolayers were lysed with distilled water. Corresponding solutions were plated on MRS, and viable adherent bacteria were counted. Adhesive capacity was determined according to the number of strains that adhered to one KB cell [41].

### Effects of lactobacilli on cytokine production by human peripheral blood mononuclear cells (PBMCs)

PBMCs were isolated from the donors’ peripheral blood by Ficoll gradient centrifugation. PBMCs (2 × 10^6^ cell/mL) were incubated at 37 °C (5% CO_2_) for 2 h and then incubated alone (the control) or with either *S*. *mutans* ATCC 25175 (10^7^ CFU/mL) or oral lactobacilli (10^7^ CFU/mL) for 12 h at 37 °C (5% CO_2_). The suspensions in the wells were aspirated and washed with RPMI 1640 medium (Thermo Fisher, Shanghai, China). For the double-challenge, *S*. *mutans* ATCC 25175-stimulated PBMCs (2 × 10^6^ cell/mL) were incubated either alone or with oral lactobacilli (10^8^ CFU/mL) for 24 h prior to cytokine analysis [11]. The production of anti-inflammatory IL-10 cytokine and pro-inflammatory IL-12p70 cytokine were determined by enzyme linked immunosorbent assay (ELISA; R&D Systems, Minneapolis, MN, USA).

### Statistical analysis

All experiments were performed in triplicate. Results were analyzed using Student’s *t*-test by SPSS 19.0. Statistical significance was set at *P* < 0.05.

## Abbreviations

CFU: Colony forming unit
DNA: Deoxyribonucleic acid
ELISA: Enzyme linked immunosorbent assay
EPS: Extracellular polysaccharides
IL: Human peripheral blood mononuclear cells cytokine interleukin
MRS: De man, Rogosa, Sharpe
PBS: Phosphate buffered saline
PYG: Peptone yeast glucose
TSB: Tryptone soy broth
